# Supplementary material for: Pulsed-electron illumination does not reduce beam damage for imaging biological macromolecules
Source: Nat Commun. 2026 Jun 17;17:7677. doi: 10.1038/s41467-026-74316-1 (PMC13433904; doi:10.1038/s41467-026-74316-1)
Supplement: Supplementary file 1 [file 41467_2026_74316_MOESM1_ESM.pdf]

Supplementary Information

Pulsed-electron illumination does not reduce beam damage for imaging biological macromolecules

Vishal Kumar[1,2], Julika Radecke[1,2], Chinmaya K.V.[1,2], Inayathulla Mohammed[1,2,4], Ricardo C. Guerrero-Ferreira[1,2,5], Daniel Harder[3], Dimitrios Fotiadis[3] and Henning Stahlberg[1,2,*]

1. Laboratory of Biological Electron Microscopy, Institute of Physics, School of Basic Sciences, EPFL, Rt. de la Sorge, 1015 Lausanne, Switzerland
2. Department of Fundamental Microbiology, Faculty of Biology and Medicine, University of Lausanne, Rt. de la Sorge, 1015 Lausanne, Switzerland.
3. Institute of Biochemistry and Molecular Medicine, University of Bern, Bühlstrasse 28, 3012 Bern, Switzerland
4. Present address: Dubochet Center for Imaging Lausanne, EPFL and UNIL, EPFL VPA DFI-Lausanne, 1015 Lausanne, Switzerland
5. Present address: Robert P. Apkarian Integrated Electron Microscopy Core, Emory University School of Medicine, 1512 Dickey Drive NE, Atlanta, GA 30322, USA

* Correspondence should be adressed to: henning.stahlberg@epfl.ch

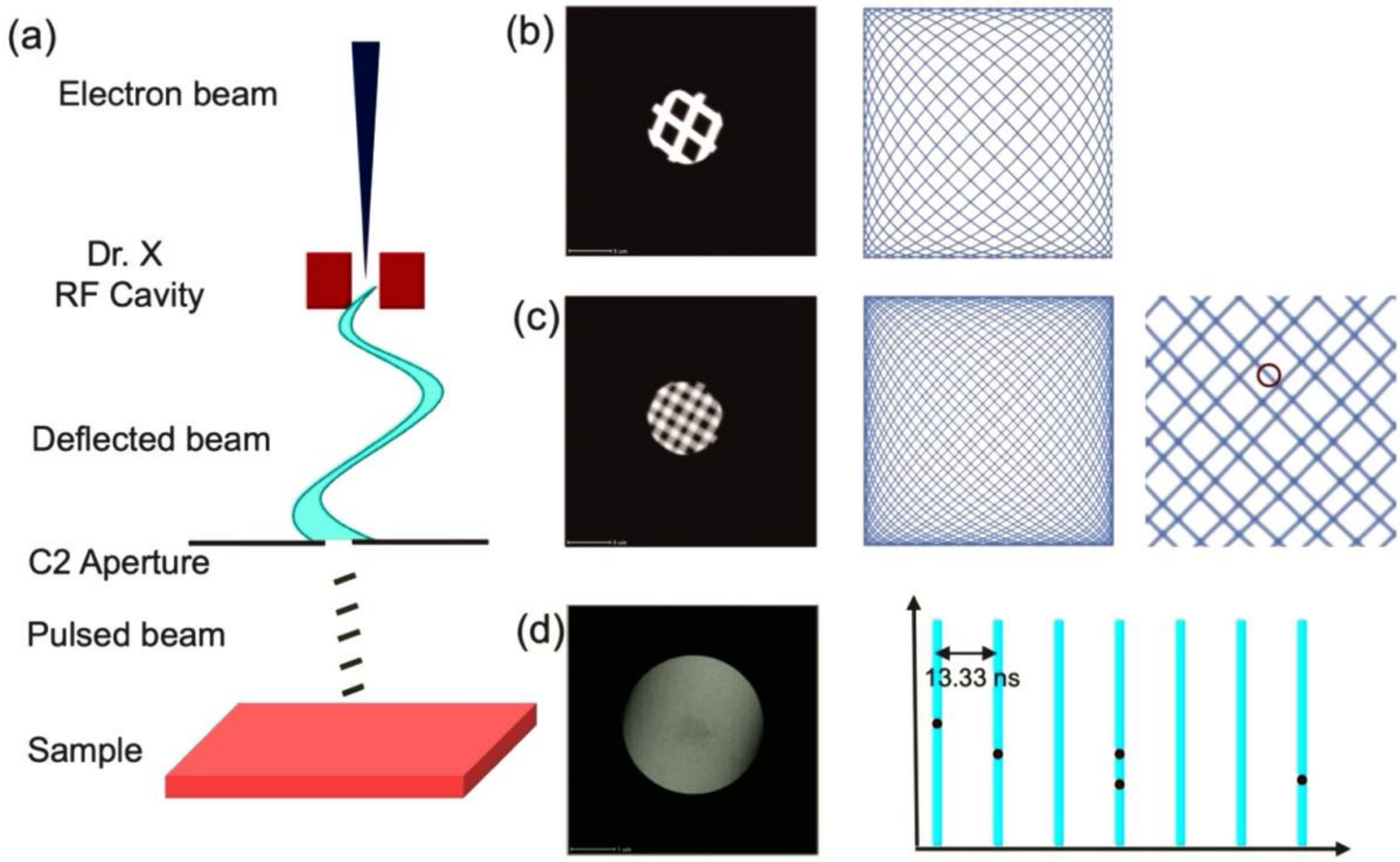

Supplementary Figure 1. The working principle of the RF cavity illumination. a Schematic for the working of RF cavity. Experimental electron beam image and simulated Lissajous patterns **b** with and **c** without phase shift showing 32×33 crossings. **d** Finally, the chopped electron beam with corresponding temporal profile, showing spacing of 13.33 ns between each pulse corresponding to difference between two RF modes (75 MHz). First the electron beam is focused onto the RF cavity, where a strong magnetic field deflect the electron-beam to form a Lissajous pattern. After that, a small phase shift is applied between the two RF modes, to separate two beams riding on top of each other. Then, using the combination of condenser aperture, spot size, illumination and magnification, the electron beam is chopped, while a homogenous electron beam covers the full aperture. Now, as the beam is making a full circle taking 13.33 ns and only a very small section of beam is able to go through the aperture, a pulsed-electron beam with temporal spacing of 13.33 ns is created.

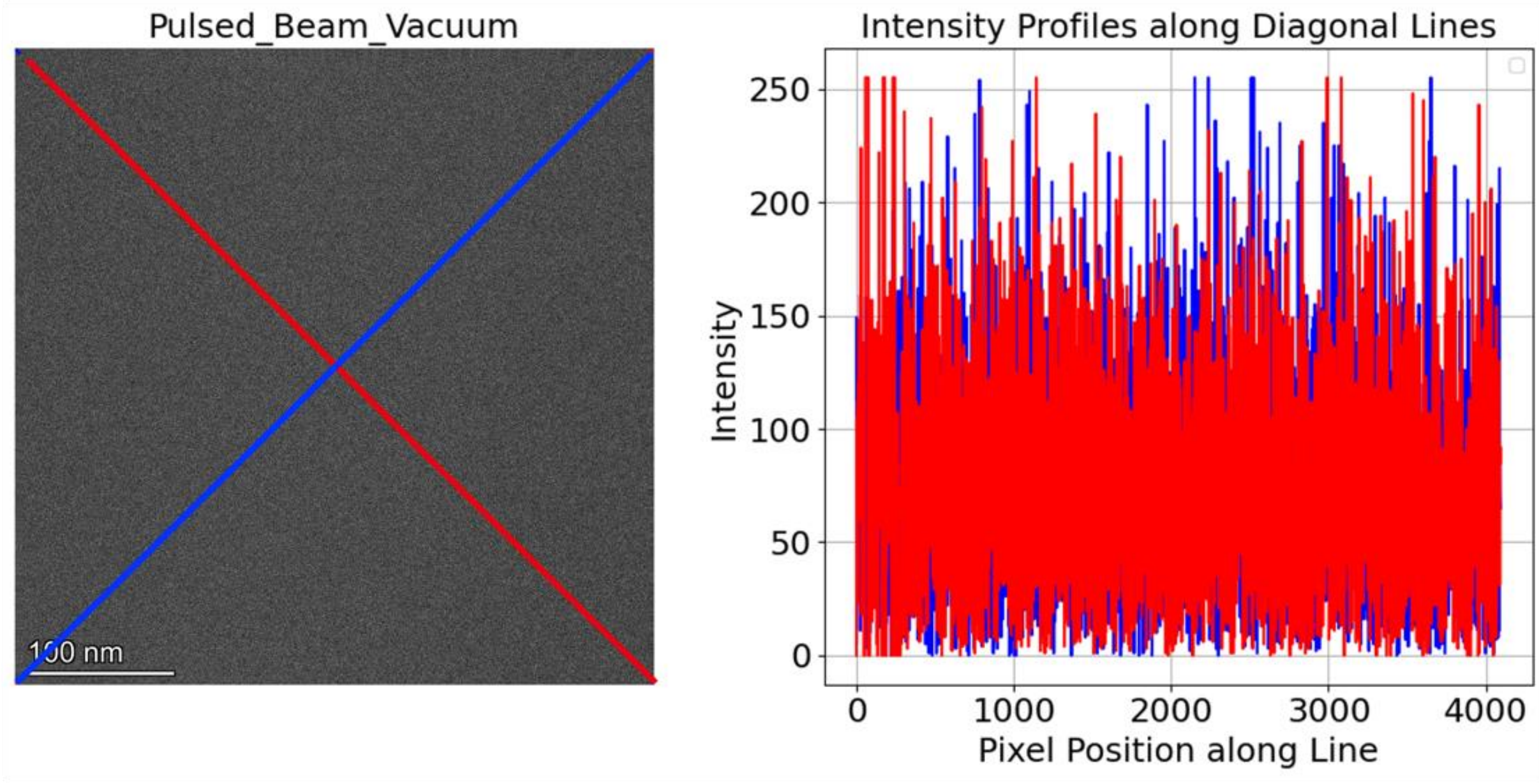

Supplementary Figure 2. TEM image over vacuum and corresponding intensity profiles along the diagonals demonstrating pulsed-beam beam homogeneity. Left: The TEM image, recorded without the presence of a sample. The blue and red diagonal lines are the traces, along which the profile was measured. Scale bar: 100 nm. Right: The two profiles along the diagonal lines indicated homogeneous illumination across the field of view. Exposure time is 2 seconds.

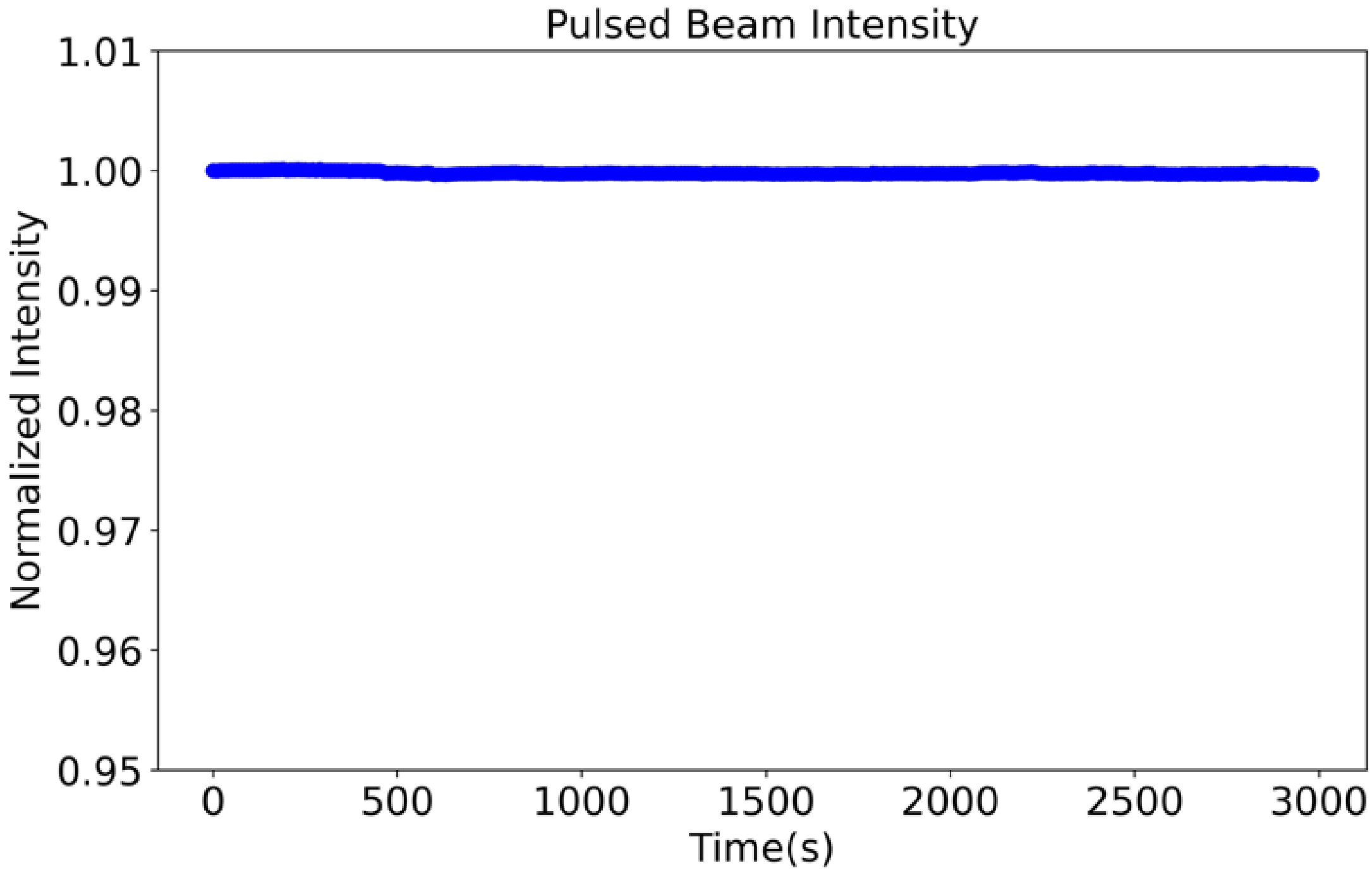

Supplementary Figure 3. Normalized intensity of the pulsed beam over time. The constant value around 1.0 documents the presences of a stable beam for 50 minutes, which was found suitable for imaging.

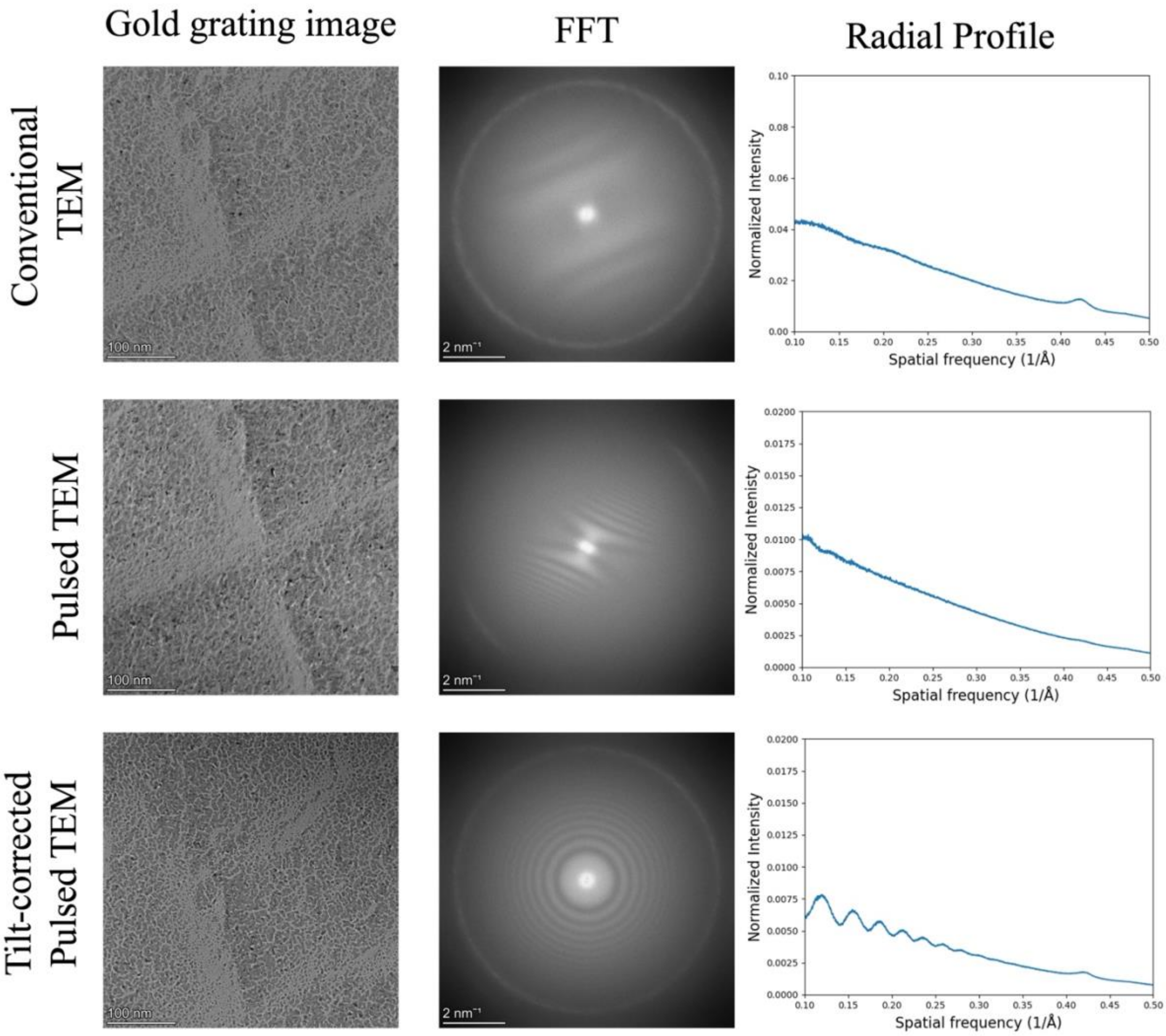

Supplementary Figure 4. TEM images of gold grating with different electron illumination and corresponding Fourier transform of images. The Fourier transforms show information transfer in different directions up to the Nyquist limit (Magnification: 75kx, Pixel size: 1.05 Å). Top row: Conventional illumination without the RF cavity in operation. Center row: The RF cavity is operating, inducing beam tilt. Bottom row: gun-tilt corrected RF cavity operation. The DrX.Works cavity induces a strong beam tilt, leading to partial information transfer (central row), which is corrected by introducing tilt in the gun (bottom row). The radial profile shows a similar performance with conventional (top) and with tilt-corrected pulsed (bottom) operation. The conventional and the tilt-corrected pulsed TEM profiles show a similar gold diffraction intensity at 0.425 $Å^{-1}$ (2.35 Å).

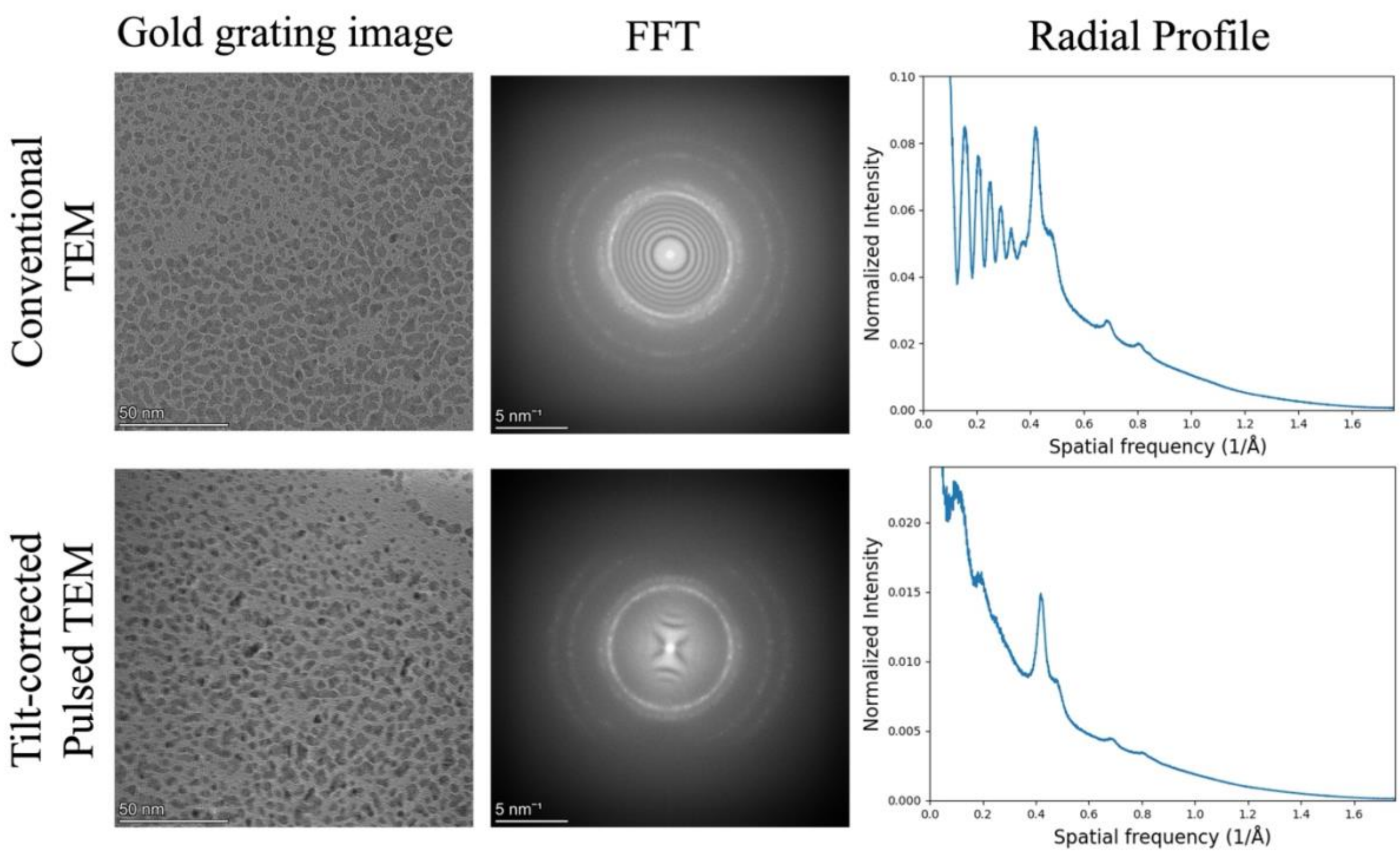

Supplementary Figure 5. High-resolution TEM images of gold grating with different electron illuminations and corresponding Fourier transforms. Top row: Conventional illumination. Bottom row: Tilt-corrected pulsed operation. Left: recorded images. Center: Fourier transforms, showing information transfer in different directions up to the Nyquist limit (Magnification: 195kx, Pixel size: 0.403 Å). Right: Radial profiles show for both, pulsed and conventional TEM illumination, information up to very high frequencies. The Image acquired with the pulsed mode shows partial loss of information in higher order diffraction rings 0.687 $Å^{-1}$ (1.45 Å), and 8.07 $Å^{-1}$ (1.24 Å), resulting from combination of increased beam tilt and loss of spatial coherence. The data for the pulsed mode were taken at lower defocus, resulting in lower intensity values for pulsed illumination.

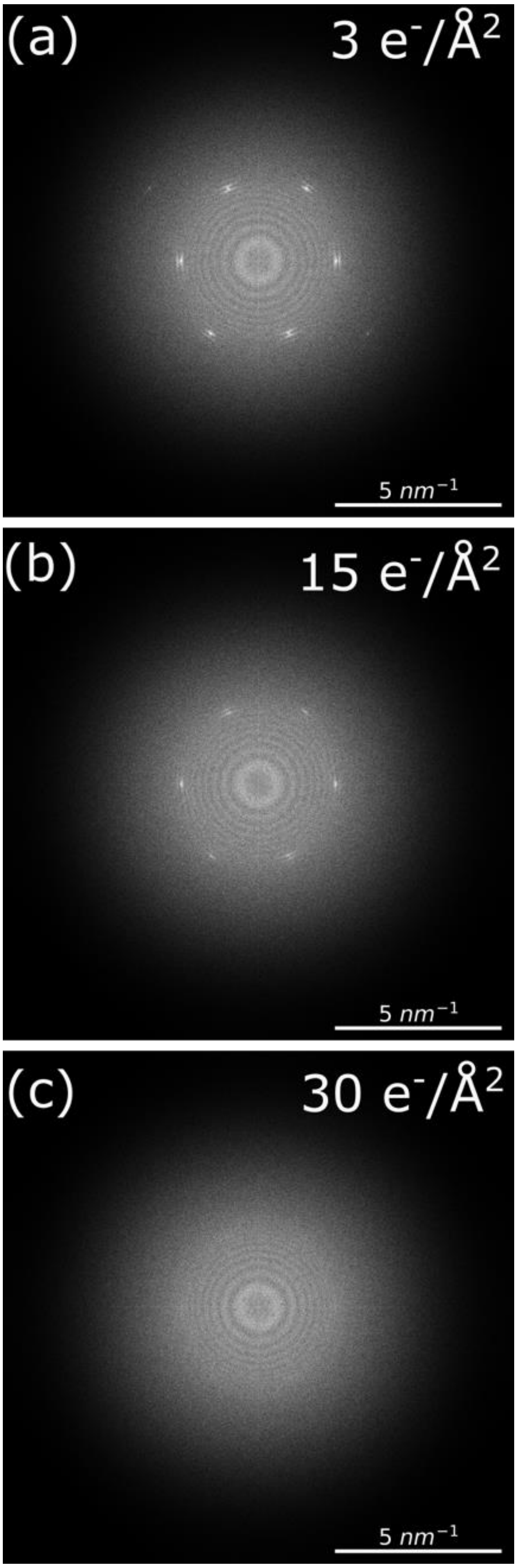

Supplementary Figure 6. Representative computed power spectra showing fading of paraffin diffraction spots with accumulated electron dose. a Power spectrum of the first recorded image. **b** Power spectrum of an image after 12 e^{-}/Å² had been given to the sample before. **c** Power spectrum of an image after 27 e^{-}/Å² had been given to the sample before.

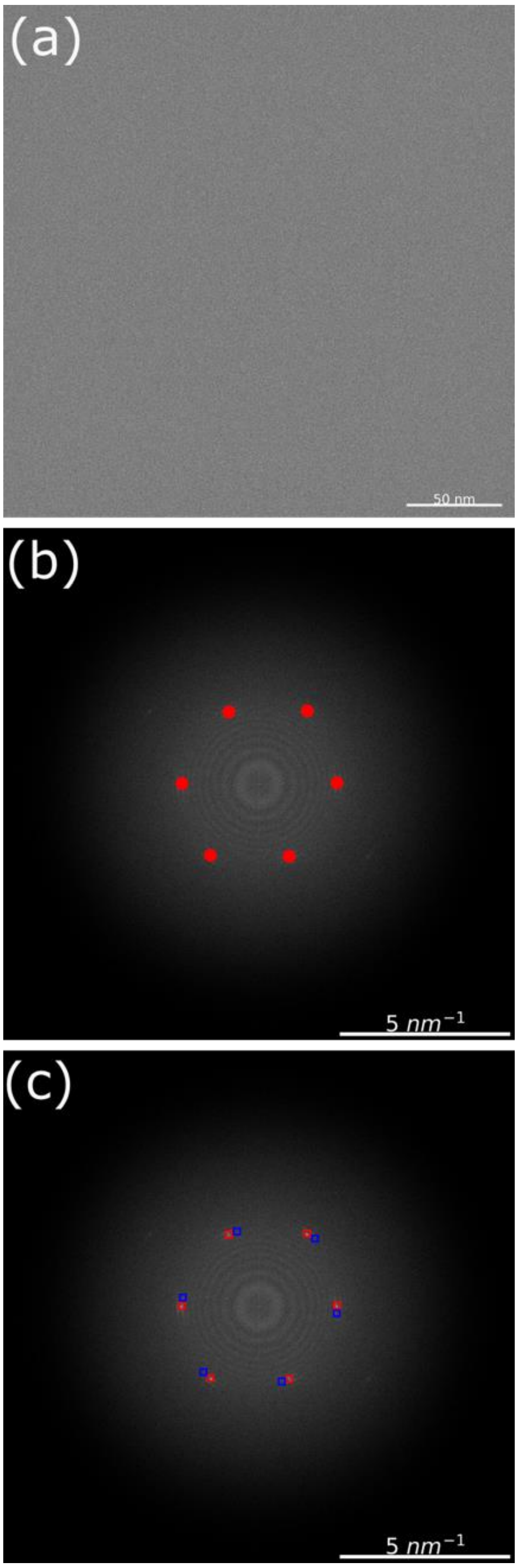

Supplementary Figure 7. Data processing pipeline for paraffin data for random and pulsed illumination. a First, the image was drift corrected using MotionCorr2 and CTF corrected (phase flipping). **b** Then, from the computed power spectrum of the image, the six innermost diffraction peaks ((110) and (200) reflections and their Friedel pairs) were identified. **c** For each peak, the intensity was integrated within a box of 50*50-pixels centered on the peak. For background subtraction, the intensity within a box of the same size but placed adjacent to the peaks was integrated, and this value was subtracted from the peak intensity. The mean of the background-subtracted intensities was then used for plotting against the cumulative electron dose.

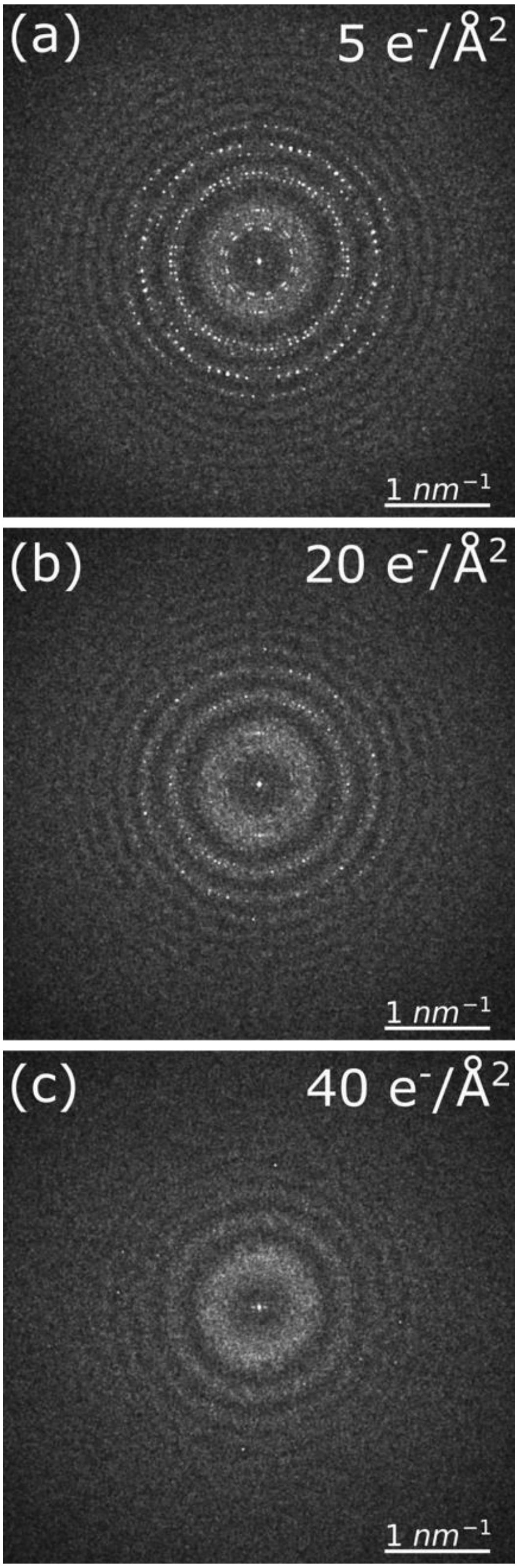

Supplementary Figure 8. Representative power spectrum showing fading of purple membrane diffraction spots with accumulated electron dose. The computed power spectra were Fourier-cropped from 4096*4096 to 1024*1024 pixels for better visualization of diffracted spots. **a** The power spectrum of the first recorded image. **b** Power spectrum after prior 15 $e^-/Å^2$. **c** Power spectrum after prior 35 $e^-/Å^2$.

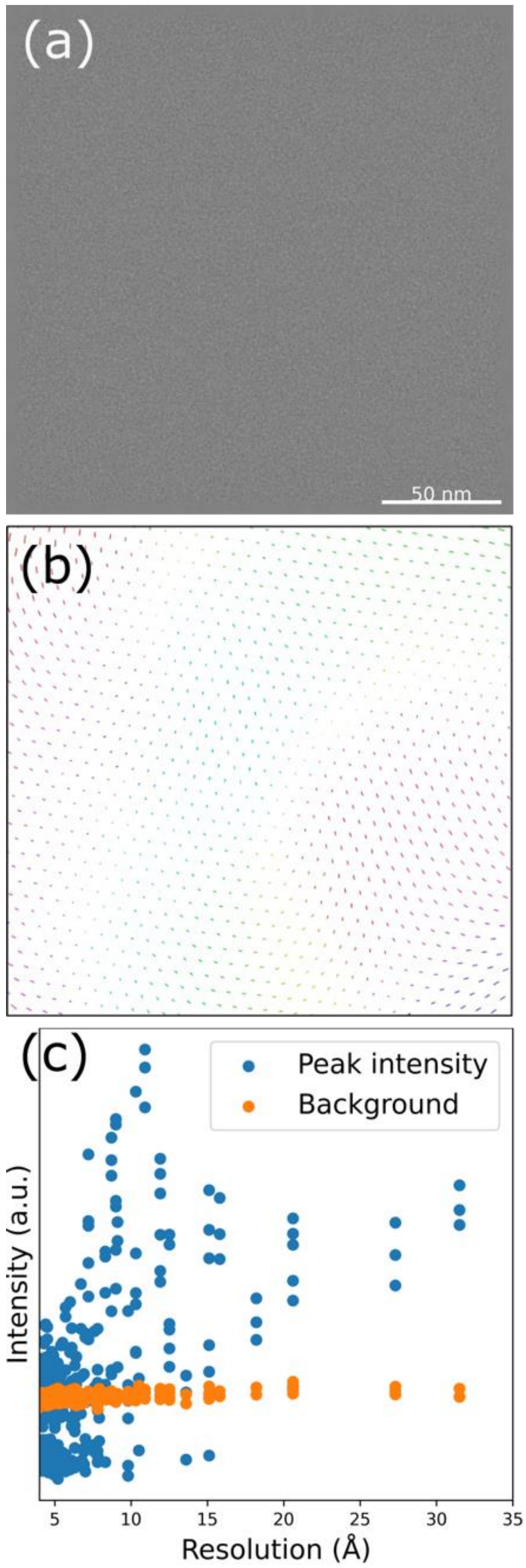

Supplementary Figure 9. Data processing pipeline for purple membrane crystal images. **a** First the image was drift corrected and CTF corrected. **b** the image was then unbent to compensate for protein crystal distortions, using the FOCUS software to enhance the intensity of the diffracted spots. The lattice unbending vectors shown here have a 10x exaggerated length for better visualization. From the FFT of the image, background-corrected peak intensities were then noted using MMBOXA script from the MRC software package, implemented in FOCUS. **c** Background-subtracted peak intensities and the background intensity surrounding each peak are plotted as a function of the peak resolution. From this, peaks were categorized into four different resolution zones (6 Å: 6-7 Å, 8 Å: 7-9 Å, 10 Å: 9-12 Å, 20 Å: 12-35 Å) for further analysis.

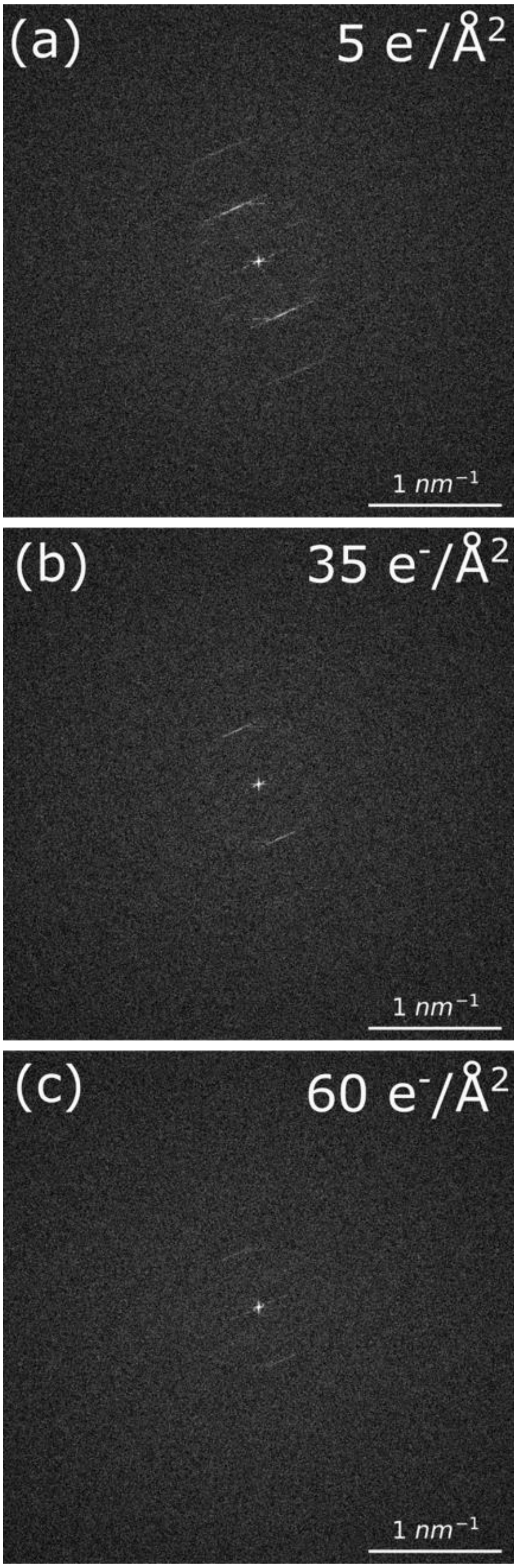

Supplementary Figure 10. Representative power spectra showing fading of diffracted rings with accumulated electron dose for TMV. The power spectra were Fourier cropped from 4096*4096 to 1024*1024 pixels for better visualization of diffracted rings. **a** Power spectrum of the first recorded image. **b** Power spectrum after prior 30 $e^{-}/Å^2$. **c** Power spectrum after 55 prior $e^{-}/Å^2$.

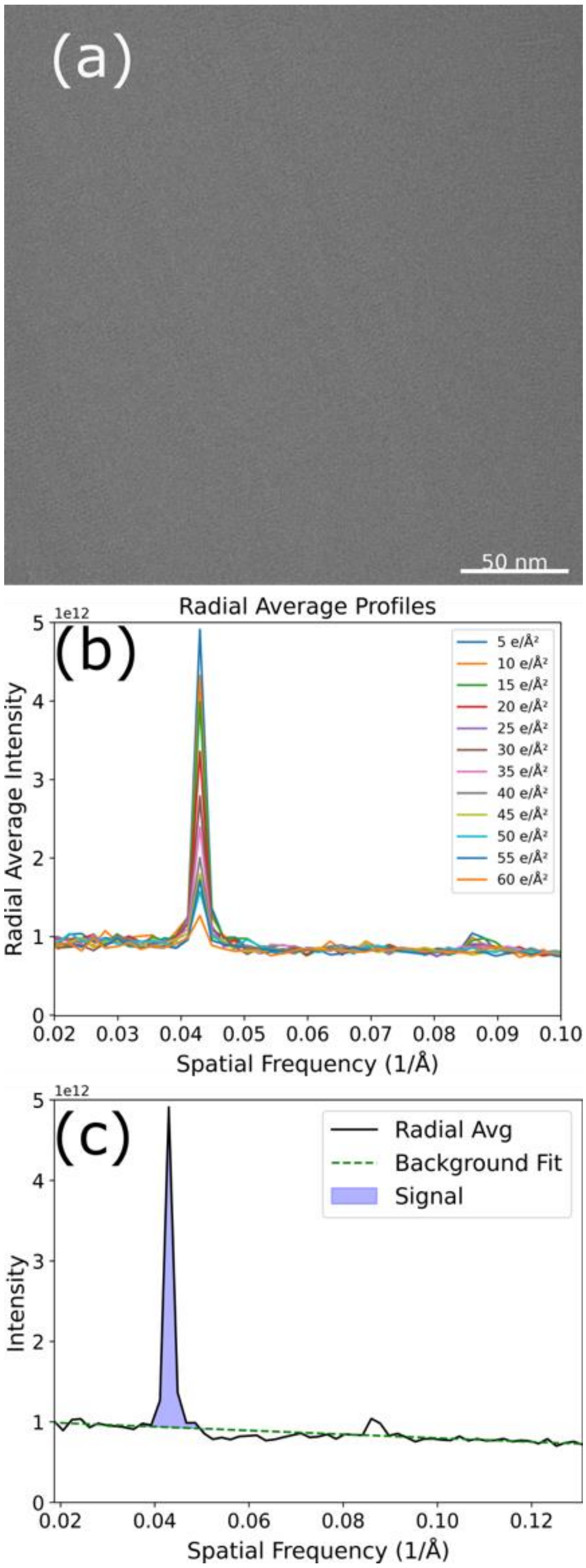

Supplementary Figure 11. Data processing pipeline for images of TMV embedded in vitreous ice. a First, images were drift corrected and CTF corrected as in previous datasets. **b** Then, from the computed power spectrum of the image, the radial average of the intensity was calculated, using a binning factor of 5 pixels. The first and second reflections at 23 and 11.5 Å resolution are discernible. **c** the background subtraction was performed and the integrated area under the first peak was measured and plotted against accumulative electron dose for 23 Å resolution peak.

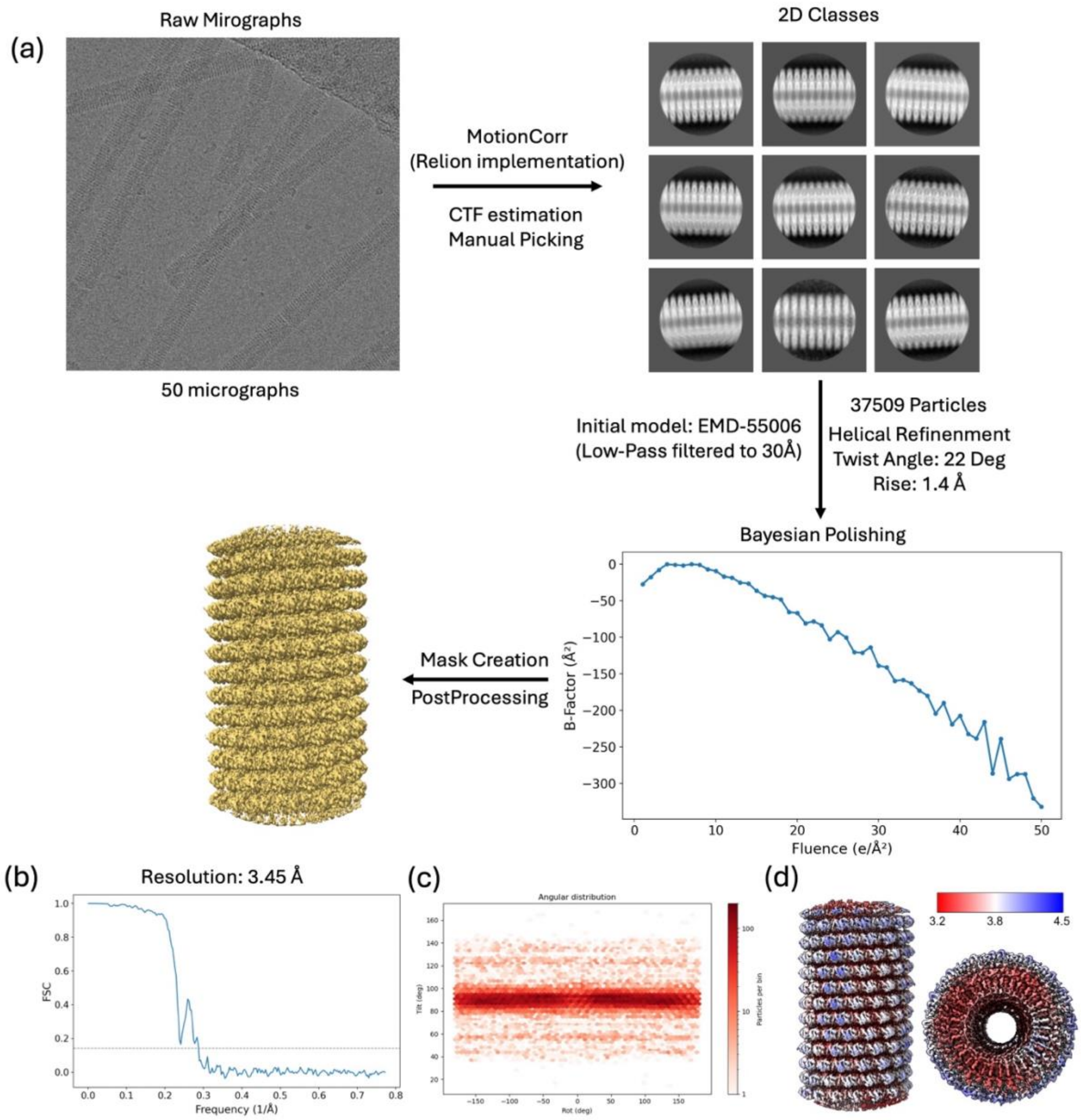

Supplementary Figure 12. Image processing of TMV images recorded with conventional illumination. (a) Pipeline for single-particle analysis (SPA) of TMV. Raw micrographs were motion-corrected using RELION's own implementation, followed by CTF estimation. Particles were picked manually and sorted by 2D classification. A subset of 37,509 particles was selected and refined by Helical Refinement with an initial twist of 22° and rise of 1.4 Å. Particles were then subjected to Bayesian polishing, followed by a second 3D refinement. A refined mask was applied, and post-processing yielded the final map shown at the end of the pipeline. **b** Fourier shell correlation (FSC) curve, **c** angular distribution of the particles contributing to the final reconstruction, and **d** local resolution map of the final TMV reconstruction.

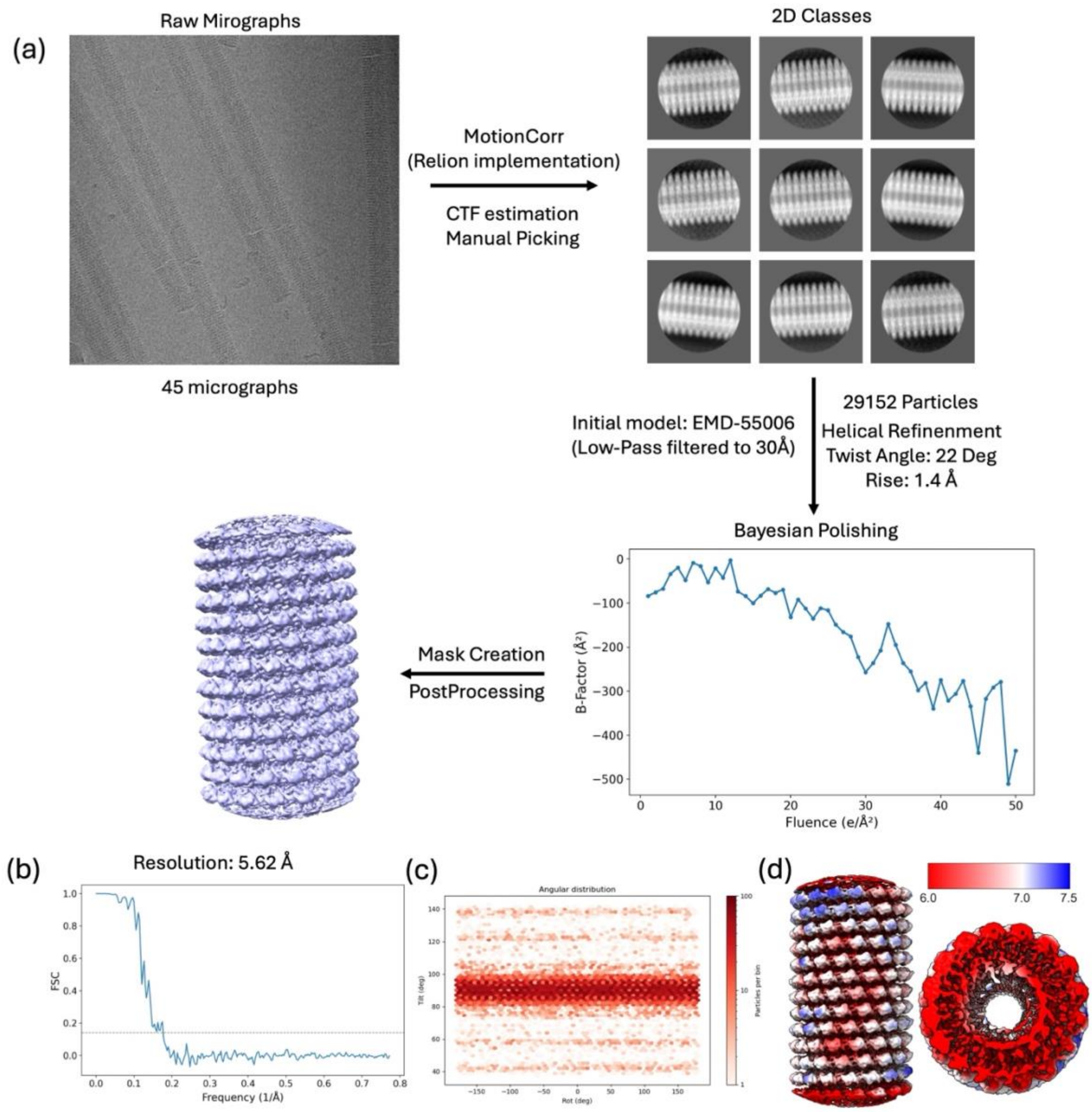

Supplementary Figure 13. Image processing of TMV images recorded with pulsed illumination. a Pipeline for single-particle analysis (SPA) of TMV. Raw micrographs were motion-corrected using RELION's own implementation, followed by CTF estimation. Particles were picked manually and sorted by 2D classification. A subset of 29,152 particles was selected and refined by Helical Refinement with an initial twist of 22° and rise of 1.4 Å. Particles were then subjected to Bayesian polishing, followed by a second 3D refinement. A refined mask was applied, and post-processing yielded the final map shown at the end of the pipeline. **b** Fourier shell correlation (FSC) curve, **c** angular distribution of the particles contributing to the final reconstruction, and **d** local resolution map of the final TMV reconstruction.

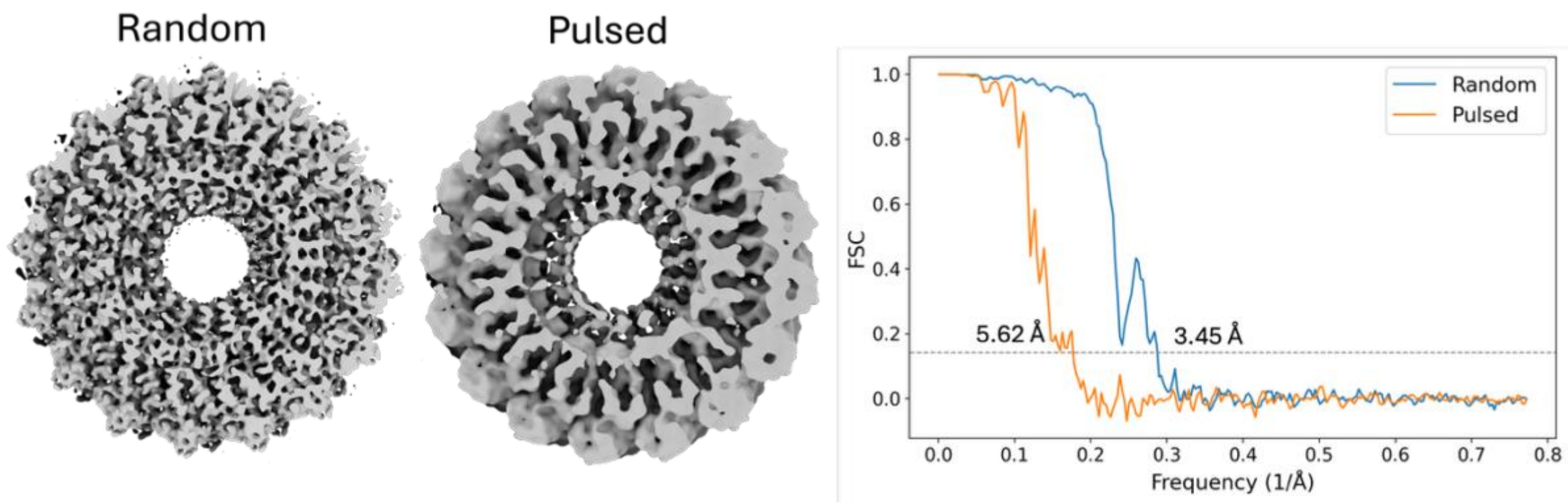

Supplementary Figure 14. Comparison of TMV reconstructions from conventional and pulsed illumination. Cross-sectional view of TMV maps for Random and Pulsed illumination and corresponding FSC plots for the TMV 3D reconstruction, comparing random with pulsed illumination. The lower resolution achieved with pulsed illumination may be due to beam stability and beam tilt issues.

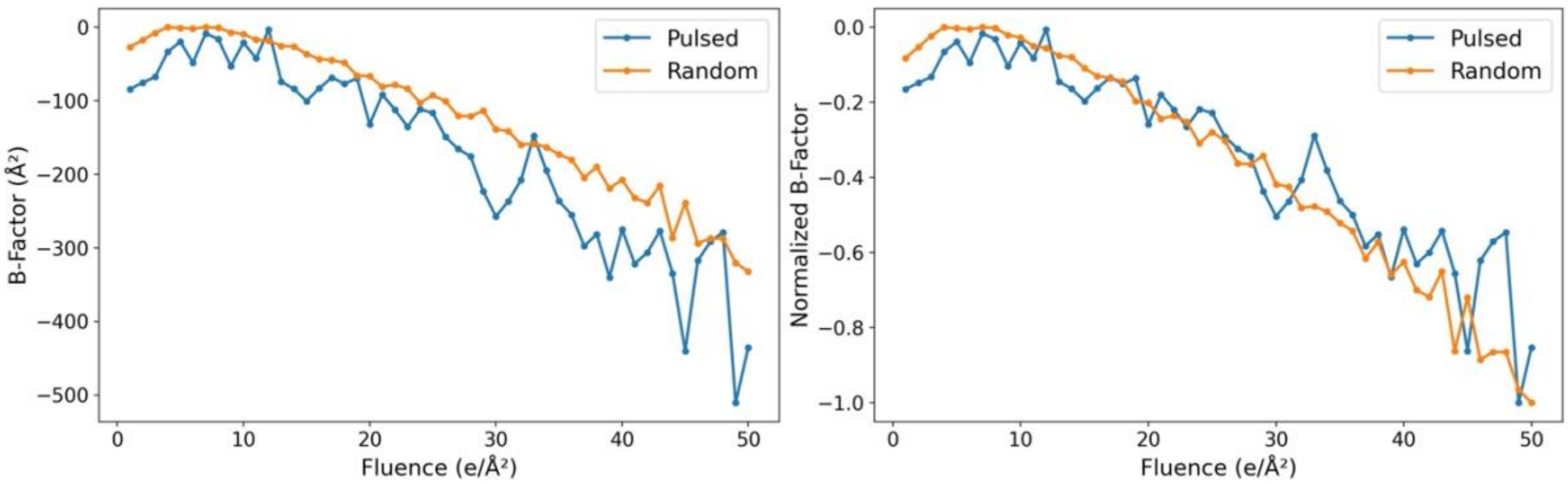

Supplementary Figure 15. Comparison of resolution loss in TMV images recorded with conventional and pulsed illumination. a Per-frame B-factors, and **b** normalized per-frame B-factors for TMV datasets, analyzed in Relion5 for both, pulsed and random electron beam illumination. B-factor values are lower and noisier for the pulsed illumination dataset, as the map resolution is lower (5.62 Å) compared to that recorded with random illumination (3.45 Å). However, the decay profiles that inform about beam damage accumulation, are similar, especially after normalization, again indicating that damage dynamics are similar for pulsed and random illumination modes.

Supplementary Table 1. Beam statistics for imaging under pulsed and random electron-beam configuration.

**Sample**	**Paraffin**		**Purple Membrane**		**TMV**	
	Pulsed	Random	Pulsed	Random	Pulsed	Random
**Pixel Size (Å)**	0.65	0.65	0.52	0.52	0.65	0.65
**Beam-Current (pA)**	4	4	3	3	3	3
**Beam Diameter (nm)**	540	540	390	390	560	560
**Dose-rate (e^-/Å^2 s)**	1.35	1.36	2.08	2.10	1.10	1.10
**Probability (1 e^-) per pulse**	23.40 %	NA	20.05 %	NA	21.34 %	NA
**Probability (2 e^-) per pulse**	3.78 %	NA	2.61 %	NA	3.02 %	NA

Supplementary Table 2. Critical dose (e^-/Å²) statistics for paraffin and TMV.

Measurement Number	Paraffin (4 Å)		TMV (23 Å)	
	Pulsed	Random	Pulsed	Random
1.	12.19	11.79	36.21	33.98
2.	9.05	12.62	35.08	30.80
3.	12.54	10.1	38.29	35.55
4.	9.13	11.33	37.86	42.90
5.	9.88	13.02	40.49	31.92
6.	12.24	11.75	37.09	39.57
7.	10.61	12.78	32.27	37.39
8.	12.31	11.53	42.45	32.62
9.	12.67	10.49	46.31	31.35
10.	10.90	11.66	35.34	36.09
**Mean ± Std**	11.15 ± 1.47	11.71 ± 0.94	38.15 ± 3.84	35.22 ± 3.70

Supplementary Table 3. Critical dose ($e^{-}/Å^{2}$) statistics for purple membrane across different resolution zones.

Measurement Number	Purple Membrane							
	(6 Å)		(8Å)		(10 Å)		(20 Å)	
	Pulsed	Random	Pulsed	Random	Pulsed	Random	Pulsed	Random
1.	13.74	15.68	26.97	17.84	16.80	16.29	29.07	22.57
2.	14.20	16.92	15.57	16.28	21.42	16.01	22.49	20.06
3.	17.02	13.57	10.91	14.52	17.78	12.38	20.99	14.94
4.	19.65	10.81	15.33	12.86	14.38	19.10	18.07	17.85
5.	18.28	23.33	15.13	15.01	14.50	19.41	24.28	15.74
6.	22.02	12.17	14.45	16.70	14.51	16.17	16.82	35.21
7.	9.61	14.04	21.04	16.05	14.80	15.97	18.31	22.22
8.	23.25	15.07	15.37	14.98	19.13	18.53	17.08	17.07
9.	14.72	11.36	17.29	13.75	14.99	16.02	20.44	21.15
10.	13.83	12.39	14.09	16.07	17.13	16.89	16.41	19.78
**Mean ± Std**	16.64 ± 4.01	14.63 ±3 .41	16.62 ± 4.21	15.41 ± 1.40	16.54 ± 2.25	16.68 ± 1.92	20.40 ± 3.80	20.66 ± 5.45

Supplementary Table 4. Cryo-EM data collection for TMV SPA analysis.

**Data collection and processing**	**Random illumination (EMDB-57764)**	**Pulsed Illumination (EMDB-57762)**
Magnification	120kx	120kx
Voltage (kV)	300	300
Electron exposure (e^{-}/Å^2)	50	50
Defocus range (µm)	1.4-1.7	1.4-1.7
Pixel size (Å)	0.6475	0.6475
Symmetry imposed	Helical	Helical
Initial particle images (no.)	37,572	29,162
Final particle images (no.)	37,509	29,152
Map resolution (Å)	3.45	5.62
Map resolution FSC threshold	0.143	0.143
