# Supplementary material for: Pulsed-electron illumination does not reduce beam damage for imaging biological macromolecules
Source: Nat Commun. 2026 Jun 17;17:7677. doi: 10.1038/s41467-026-74316-1 (PMC13433904; doi:10.1038/s41467-026-74316-1)
Supplement: Supplementary file 3 [file 41467_2026_74316_MOESM3_ESM.pdf]

nature portfolio

Peer Review File

Pulsed-electron illumination does not reduce beam damage for imaging biological macromolecules

Corresponding Author: Professor Henning Stahlberg

This file contains all reviewer reports in order by version, followed by all author rebuttals in order by version.

Version 0:

Reviewer comments:

Reviewer #1

(Remarks to the Author)
This is a very important and timely study that explores the use of pulsed electron illumination in modern cryo-EM microscopes. To my knowledge, this represents the first demonstration of pulsed illumination applied to vitrified samples, providing proof-of-concept evidence that such approaches are feasible with state-of-the-art instrumentation. More importantly, this study is noteworthy because its findings directly contradict previous speculation regarding the mitigation of radiation damage in cryo-EM under pulsed illumination conditions.

The results are clearly presented and consistent with the authors' conclusions. The data support the main claims, though additional discussion and detail would provide valuable guidance for the community. Furthermore, a discussion of why single-particle analysis (SPA) reconstructions are not included in the current work—especially if there are specific challenges associated with applying SPA under pulsed illumination—would further strengthen the manuscript. SPA could provide more systematic information on radiation damage in cryo-EM, so either including such an analysis or explaining the challenges that prevent it would add valuable context.

In conclusion, this study provides the first implementation of pulsed electron illumination applied to biological samples including vitrified specimens, directly challenging the speculation that pulsed illumination could inherently mitigate radiation damage. Although the work is not exhaustive, it nonetheless establishes a crucial proof of concept on state-of-the-art instrumentation. By opening this new experimental space, the authors lay an important foundation for future studies, on whether and how to proceed with pulsed illumination in cryo-EM.

Technical details:
1. Random illumination = conventional illumination; this note should be stated earlier in the paper, including in the abstract.
2. Page 2, paragraph 2 discusses acceleration voltage vs. radiation damage but is missing a conclusion such as "no optimal acceleration voltage will satisfy a range of different sample thicknesses in terms of lowering radiation damage."
3. Page 2, paragraph 3 discusses that the previous work was acquired with very low dose, which is not "practical for data acquisition strategies"; it would also be nice to mention that data analysis for cryo-EM is another big challenge at this extremely low dose.
4. Fig. 1c shows the temporal profiles of the pulsed beam and random beam, but the figure caption says something different.
5. For Fig. 1b,c, it would be nice to specify if the panels are experimental data or illustration diagrams.
6. Page 5, paragraph 1 states that the 50 µm C2 aperture is used as a selective aperture to allow the "desired portion" of the beam to pass. It would be helpful to clarify what is considered "desired." Since Fig. 1a,b shows no lens between the Lissajous pattern and the condenser aperture, a brief explanation of how a 50 µm aperture can capture only part of a ~2–3 µm wide pattern (as in Fig. 1b) would improve clarity. I may have misinterpreted the way the C2 aperture is used to "select" part of the beam, so a short clarification from the authors would be useful.
7. Can the exposure time be specified for Supplementary Figure 1?
8. Page 5, paragraph 2: Are Supplementary Figures 2 and 3 supposed to be swapped?
9. Page 5, paragraph 2: I am not finding Supplementary Table 1.
10. Page 5, paragraph 2: "…Supplementary Figure 2 shows 'high-resolution' is not 'hindered.'" A comparison of Thon ring radial profiles between pulsed and conventional illumination would better demonstrate this point. A quantitative radial profile would be more convincing than image inspection alone. This seems especially relevant given the need to tilt the gun to compensate for the RF cavity.

11. Figs. 2–4: For all the peak intensity vs. fluence plots, pulsed illumination and conventional illumination dose series are normalized with respect to the first images, respectively. Can the authors discuss the absolute intensity of the peaks? Are they comparable?
12. Figs. 2–4: The authors conclude no significant differences in critical doses for pulsed and conventional illumination, but multiple plots (Figs. 2c, 3c, 4c) suggest that the critical dose spread (i.e., error bars) is larger for pulsed illumination; it would be nice to comment on that.
13. Fig. 4: TMV is the only vitrified biological sample analyzed, and only one peak at low resolution (23 Å) is shown. Given the importance of this sample, it would be helpful to explain why SPA analysis was not performed, since that could provide a more comprehensive view across a range of frequencies.
14. Conclusion, paragraph 2: This study uses a much longer pulse interval (13.33 ns vs. hundreds of ps in prior work) and a much higher accumulated dose (tens of $e^-/Å^2$ vs. 0.1 $e^-/Å^2$). It would be valuable to discuss how dose rate, accumulated dose, and pulse repetition rate interact, and whether an optimal balance might exist.
15. It would be helpful for the authors to provide a hypothesis why the previously reported advantages of pulsed illumination are not observed in this case, to clarify how their results relate to prior studies.
16. Please include EMPIAR deposition of the raw data for validation purposes.

(Remarks on code availability)
The code is concise and clear and appears to provide a transparent, end-to-end analysis pipeline. However, not finding the raw images, I was unable to run the code to verify its functionality.

Reviewer #2

(Remarks to the Author)
The authors have presented a very important study of radiation damage in cryoEM, comparing an RF pulsed electron source to a continuous source of the same flux for biological specimens at liquid nitrogen temperatures. The work is well done and convincing, and will be very important to the field. I have only a few minor comments.

1. In figure 1, the scale bar indicates that the electron lissajous pattern covers an area of about 2.3 x 3 micrometers. In the text on page 5 it says that a 50 micrometer aperture was inserted downstream of the raster pattern to chop the beam. How is this possible? Is the patten magnified before the aperture? Please indicate on the pattern in the figure how large the aperture that cuts it actually is. Please also clarify how the lissajous pattern was measured.

2. On page 5 it says that 75% of the bunches were empty. Is this controlled only by the size of the aperture or also by the RF modulation parameters? Please explain how this ratio is controlled in detail.

3. The performance of the TEM without the RF enabled is far below what would be expected for a normal Krios with a cFEG, as indicated by the tests with a cross grating in Supplementary figure 2; compare this to the performance seen in ref 25. Please explain why the performance is so much worse?

Minor:
Perhaps "limiting" instead of "hindering" in the first sentence of the abstract.

(Remarks on code availability)
-

Reviewer #3

(Remarks to the Author)
This paper presents a systematic study of radiation damage on paraffin crystals, purple membrane crystals, and TMV crystals using a radio-frequency (RF) driven 300 kV Titan Krios microscope equipped with a cold field emission gun (c-FEG) to generate highly regular pulsed electron beams. The results demonstrate that pulsed-electron illumination does not reduce beam damage in imaging biological macromolecules. Overall, the experiments are well-designed and rigorous, and the conclusions provide valuable insights into the ongoing debate regarding whether pulsed illumination can mitigate sample radiation damage. However, the following issues should be reasonably resolved or explained before publication.

1) In Figure 1 of the manuscript, the authors only provide a schematic diagram of the TEM setup with brief descriptions. Could they supplement some experimental images or data to better illustrate the differences between the pulsed and random illumination modes?

2) On page 5 of the manuscript, the authors state: “It is important to note that under typical experimental conditions, approximately 75% of the electron bunches were empty, while only about 25% contained single-electron events, as detailed in Supplementary Table 1.” However, I could not find Supplementary Table 1 in the Supplementary Information.

3) Also on page 5, the authors mention: “For comparison, the temporal profile of a conventional electron beam is also shown, which was obtained by switching off the RF cavity and adjusting the condenser lens settings so that the same beam brightness and diameter was achieved.” It’s not sure whether “the temporal profile of a conventional electron beam” shown here refers to the content in Figure 1c. The authors should clearly indicate this, and clarify whether the result is experimental

or theoretical.

4) The authors should label the (1,1) and (2,1) planes in Figure 2a to facilitate reader interpretation. Similar labeling should be applied in Figures 3 and 4.

5) In Figures 2, 3, and 4, the authors present numerous curves with error bars. When stating the Ne values obtained from these curves (e.g., 11.81 e−/Å², 12.19 e−/Å², 38.15 e−/Å², 35.22 e−/Å²), the error range should be included. Furthermore, the authors mention "The radiation damage profiles, obtained from 10 independent measurements under both pulsed and random illumination conditions." I suggest that the statistical data for each independent measurement be tabulated in the Supplementary Information.

6) For paraffin crystals, the method for quantifying diffraction intensity is clear. However, for the purple membrane and TMV polycrystals shown in Figures 3 and 4, it is unclear how the diffraction intensities were extracted and calculated under segmented conditions. Could the authors provide more detailed explanations?

7) The authors note: "In our study, the interval between consecutive pulses was 13.33 ns, substantially longer than the 160 ps used by Kisielowski et al.18 or the 192 ps used by Choe et al.19." However, Flannigan et al. reported that radiation damage mitigation was observed at intervals of 5–20 µs between consecutive pulses. Did the authors conduct comparative experiments using different pulse intervals? What is their perspective on the influence of pulse interval on sample radiation damage?

8) The authors need to revise Ref. 20 in the manuscript to cite its formal version (The Innovation Life 3:100145).

9) Based on their experiments, the authors ultimately conclude that "Pulsed-electron illumination does not reduce beam damage for imaging biological macromolecules." Regarding this controversial issue, have the authors discussed the underlying mechanisms responsible for their experimental results? I suggest the authors provide some reasonable explanations and theoretical analysis for these findings, rather than solely presenting experimental conclusions.

(Remarks on code availability)

Version 1:

Reviewer comments:

Reviewer #1

(Remarks to the Author)
The revision resolved earlier ambiguities and provided details explanations of how the RF cavity selects signal, and provided informative summary of previous pulsed illumination work. It also provided important SPA metrics, including B-factors, and the discussion of the challenges in acquiring SPA data is particularly informative for guiding future work. Only minor comments for the authors to consider address:
Supplementary Figure 5 is to support that "the RF cavity has no significant negative impact on the microscope's performance" but the absolute value of the signals in the two radial profiles seem to be different enough that need an explanation, for example the signal at 0.487 Å-1 for conventional illumination >~0.08 but that of pulsed illumination is ~0.015.

(Remarks on code availability)

Reviewer #2

(Remarks to the Author)
The authors have made many improvements to the manuscript that are much appreciated. This is an important paper.

There is one serious error remaining though. The new supplementary figure 5 now presents an excellent comparison of the effect of the pulsed vs. conventional TEM. It is clear from the FFTs (loss of Thon rings at high resolution and removal of gold rings along one axis) that there is severe degradation in the spatial coherence caused by RF cavity. This is as expected (by me, anyhow) and exactly the opposite of the statement "demonstrating that the RF cavity has no significant negative impact on the microscope's performance." in the caption. Please correct this and include a description of the loss of spatial coherence and increased tilt in the text. I do not think this changes any of the conclusions regarding the radiation damage measurements in the rest of the paper but it is essential to include this as part of evaluating the effect of the RF cavity on the source and imaging performance.

(Remarks on code availability)

Reviewer #3

(Remarks to the Author)
The authors have responded to my comments. Changes have been made to manuscript and supporting information in light of my comments, which improve the quality of paper. As such, I recommend publication in Nature Communications with further editorial check.

By the way, I noticed a possible error in the newly revised manuscript. Please pay attention to correcting it by the authors: Regarding the data provided in Supplementary Table 2, I observed that the average critical dose of Paraffin in the Pulsed and Random modes should be 11.15 and 11.71 respectively, rather than the 11.81 and 12.25 mentioned in the current text. Please carefully check the data in the manuscript.

(Remarks on code availability)

Version 2:

Reviewer comments:

Reviewer #2

(Remarks to the Author)
The authors have again improved the manuscript. The beam alignments in Fig S4 are still not optimal in either case, making comparison difficult, but the new figure S5 makes it clear how much is lost. It is now good enough for publication.

(Remarks on code availability)

Open Access This Peer Review File is licensed under a Creative Commons Attribution 4.0 International License, which permits use, sharing, adaptation, distribution and reproduction in any medium or format, as long as you give appropriate credit to the original author(s) and the source, provide a link to the Creative Commons license, and indicate if changes were made.
In cases where reviewers are anonymous, credit should be given to 'Anonymous Referee' and the source.
The images or other third party material in this Peer Review File are included in the article's Creative Commons license, unless indicated otherwise in a credit line to the material. If material is not included in the article's Creative Commons license and your intended use is not permitted by statutory regulation or exceeds the permitted use, you will need to obtain permission directly from the copyright holder.
To view a copy of this license, visit https://creativecommons.org/licenses/by/4.0/

REVIEWER COMMENTS

Reviewer #1 (Remarks to the Author):

This is a very important and timely study that explores the use of pulsed electron illumination in modern cryo-EM microscopes. To my knowledge, this represents the first demonstration of pulsed illumination applied to vitrified samples, providing proof-of-concept evidence that such approaches are feasible with state-of-the-art instrumentation. More importantly, this study is noteworthy because its findings directly contradict previous speculation regarding the mitigation of radiation damage in cryo-EM under pulsed illumination conditions.

The results are clearly presented and consistent with the authors' conclusions. The data support the main claims, though additional discussion and detail would provide valuable guidance for the community.

Furthermore, a discussion of why single-particle analysis (SPA) reconstructions are not included in the current work—especially if there are specific challenges associated with applying SPA under pulsed illumination—would further strengthen the manuscript. SPA could provide more systematic information on radiation damage in cryo-EM, so either including such an analysis or explaining the challenges that prevent it would add valuable context.

We thank this reviewer for the encouraging comments. Single Particle Analysis (SPA) was approached, but we faced significant hurdles. Acquiring large datasets of TMV for SPA analysis has proven very difficult due to combination of requirement of flashing the CFEG tip (which normalizes electron optics), inability to change magnifications in big-steps (required for automated data acquisition for performing Eucentric height, finding holes etc.), and inability to change defocus values by big steps as it introduces beam tilt (required to filling in zeroes in CTF of micrographs).

Neverthless, we acquired a small dataset of TMV for SPA and the results have been documneted in **Supplementary Figure 12 and 13**. The per-frame B-factor curves show that the damage dynamics is almost similar for pulsed and random illumination mode, thus further strengthning our argument that pulsed electron beam dosen't lead to significant improvement in damage reduction for imaging biological macromolecules.

In conclusion, this study provides the first implementation of pulsed electron illumination applied to biological samples including vitrified specimens, directly challenging the speculation that pulsed illumination could inherently mitigate radiation damage. Although the work is not exhaustive, it nonetheless establishes a crucial proof of concept on state-of-the-art instrumentation. By opening this new experimental space, the authors lay an important foundation for future studies, on whether and how to proceed with pulsed illumination in cryo-EM.

We thank the reviewer for these positive comments.

Technical details:

1. Random illumination = conventional illumination; this note should be stated earlier in the paper, including in the abstract.

We have adopted the manuscipt accordingly. These changes can be found in line 31.

2. Page 2, paragraph 2 discusses acceleration voltage vs. radiation damage but is missing a conclusion such as "no optimal acceleration voltage will satisfy a range of different sample thicknesses in terms of lowering radiation damage."

We thank the reviewer for this excellent suggestion, which we have now inlcuded in the manuscript in line 61-64.

3. Page 2, paragraph 3 discusses that the previous work was acquired with very low dose, which is not "practical for data acquisition strategies"; it would also be nice to mention that data analysis for cryo-EM is another big challenge at this extremely low dose.

Keeping reviewer comments in mind, we have added additional discussion in the revised manuscript, which can be found in lines 71-72.

4. Fig. 1c shows the temporal profiles of the pulsed beam and random beam, but the figure caption says something different.

We thank the reviewer for bringing this to out attention. The caption has been modified in the revised manuscript to clearly depict the figure 1c, in line 135-138.

5. For Fig. 1b,c, it would be nice to specify if the panels are experimental data or illustration diagrams.

Keeping reviewer comments in mind, we have modified the caption of Figure 1 to address reviewer comments. We have also added a new Supplementary Figure 1, to better demonstrate the working of RF cavity.

6. Page 5, paragraph 1 states that the 50 µm C2 aperture is used as a selective aperture to allow the "desired portion" of the beam to pass. It would be helpful to clarify what is considered "desired." Since Fig. 1a,b shows no lens between the Lissajous pattern and the condenser aperture, a brief explanation of how a 50 µm aperture can capture only part of a ~2–3 µm wide pattern (as in Fig. 1b) would improve clarity. I may have misinterpreted the way the C2 aperture is used to "select" part of the beam, so a short clarification from the authors would be useful.

We thank the reviewer for this comment. As requested, we have added a small discussion to clarify this in the revised manuscript, which can be found in lines 158-162.

7. Can the exposure time be specified for Supplementary Figure 1?

We have added the exposure time for the Supplmentary Figure 1 in the revised manuscript.

8. Page 5, paragraph 2: Are Supplementary Figures 2 and 3 supposed to be swapped?

We thank the reviewer for bringing this to our attention. We have changed it to proper order in the revised manuscript.

9. Page 5, paragraph 2: I am not finding Supplementary Table 1.

We thank reviewer to bringing this to our attention. We have added the Supplementary Table 1 correctly in the revised manuscript.

10. Page 5, paragraph 2: "…Supplementary Figure 2 shows 'high-resolution' is not 'hindered.'" A comparison of Thon ring radial profiles between pulsed and conventional illumination would better demonstrate this point. A quantitative radial profile would be more convincing than image inspection alone. This seems especially relevant given the need to tilt the gun to compensate for the RF cavity.

We thank the reviewer for bringing this to our attention. We have added a quantitative radial profile for conventional, pulsed and tilt-free pulsed illumination in the revised the Supplmentary Information.

11. Figs. 2–4: For all the peak intensity vs. fluence plots, pulsed illumination and conventional illumination dose series are normalized with respect to the first images, respectively. Can the authors discuss the absolute intensity of the peaks? Are they comparable?

We thank the reviewer for the critical observation. We observed no significant difference in the absolute intensities for paraffin and TMV sample, as shown in **Figure 1** below. However, for purple membrane we observed a trend, where at higher resolution (6, 8 and 10 Å) conventional illumination have higher intensity compared to pulsed mode. However, at lower resolutions (20 Å), they seem to have almost same intensity, as shown in **Figure 2** attached with this comment. Despite the different resolutions reached, the absolute intensity follows the same damage profile in both illumination cases, thus demonstrating that damage dynamics is same for pulsed and random illumination modes.

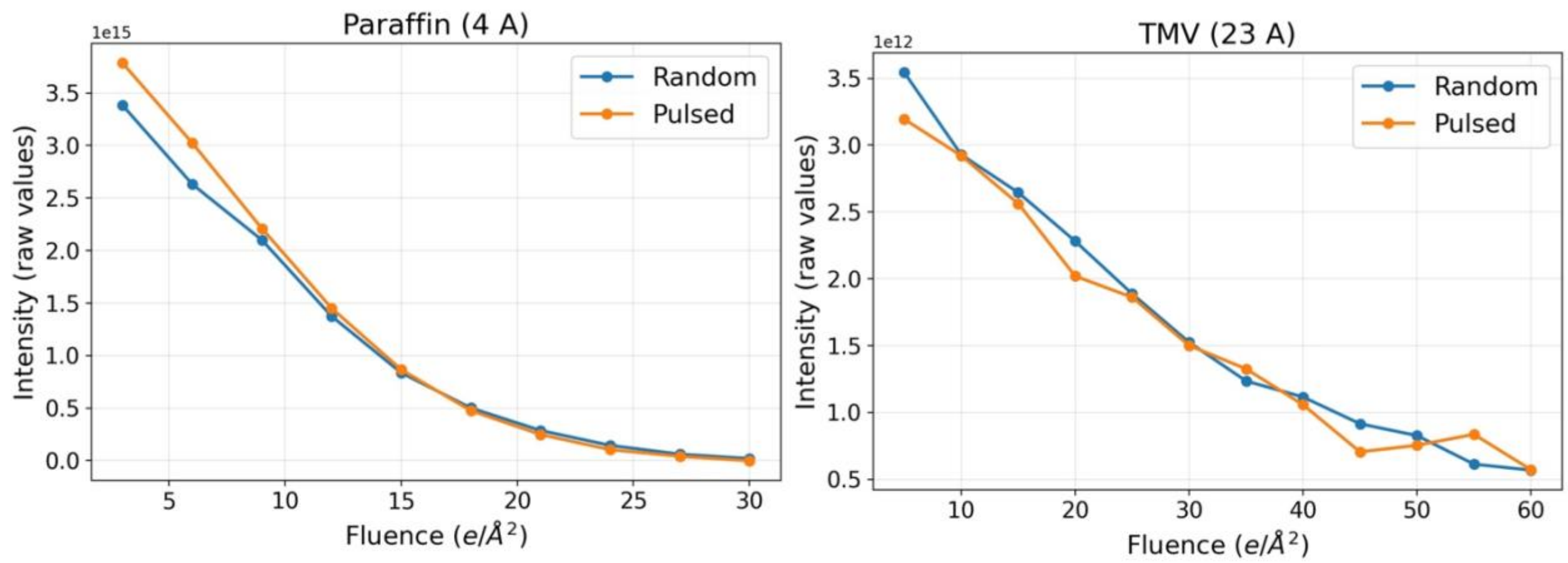

Figure 1. Absolute intensity values for paraffin and TMV sample.

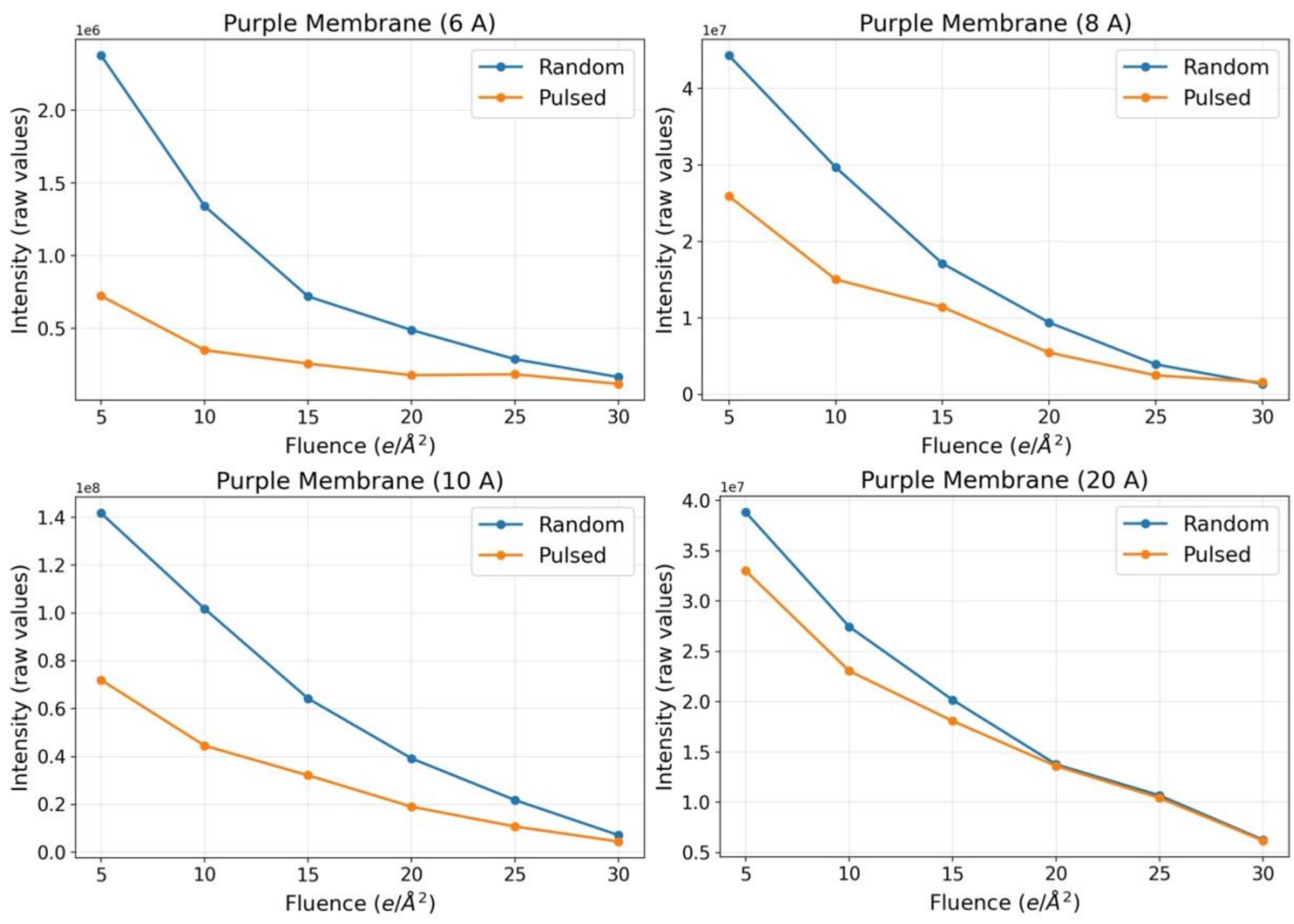

Figure 2. Absolute intensity values for purple membrane across different resolution zones.

12. Figs. 2–4: The authors conclude no significant differences in critical doses for pulsed and conventional illumination, but multiple plots (Figs. 2c, 3c, 4c) suggest that the critical dose spread (i.e., error bars) is larger for pulsed illumination; it would be nice to comment on that.

We thank the reviewer for bringing this to our attention. Supplementary Table 2 and 3, shows the actual values for critical dose, for each measurement. We agree with reviewer observation that, pulsed illumination has wider spread for critical doses. But we don't have any explanation for this trend, as this might be due to variation of carbon film thickness, some contamination build-up or something else.

13. Fig. 4: TMV is the only vitrified biological sample analyzed, and only one peak at low resolution (23 Å) is shown. Given the importance of this sample, it would be helpful to explain why SPA analysis was not performed, since that could provide a more comprehensive view across a range of frequencies.

We thank the reviewer for the valuable comments. As mentioned above acquiring large dataset with pulsed mode for SPA has proven difficult. But we have acquired small dataset, which is displayed in **Supplementary Figure 12** and **13**. The per-frame B-factor plot shows that both pulsed and random illumination has similar decay profile. Thus, it furthers strengthen our argument that pulsed electron beam doesn't significantly improve damage dynamics for imaging biological macromolecules. A small discussion has also been added in main manuscript which can be found in lines 258-264.

14. Conclusion, paragraph 2: This study uses a much longer pulse interval (13.33 ns vs. hundreds of ps in prior work) and a much higher accumulated dose (tens of $e^-/Å^2$ vs. 0.1 $e^-/Å^2$). It would be valuable to discuss how dose rate, accumulated dose, and pulse repetition rate interact, and whether an optimal balance might exist.

We thank the reviewer for the valuable insight. We have modified the manuscript in different locations, as in line 69-72, 74-80 and 84-86.

Briefly, as discussed in a previous study by Flannigan et al., it is generally believed that increasing the time between electron pulses enhances damage mitigation by allowing sufficient time for energy dissipation. In our study, the interval between pulses is 13.33 ns—longer than that used by Kisielowski et al.—yet we still do not observe any reduction in damage. Flannigan et al. used much longer pulse intervals (5, 20, and 100 µs), but this results in extremely slow dose delivery. For example, using the dose rate reported by Flannigan et al., it would take approximately 2.78×10^5 s (more than three days) to acquire a single image with a dose of 50 $e^-/Å^2$, which is not practical.

Even with current advances in high-brightness CFEGs that allow operation at higher beam currents, increasing the time between incoming pulses would still lead to unacceptably long acquisition times for a single micrograph. In practical cryo-EM workflows, hundreds to thousands of micrographs must be collected, making the approach demonstrated by Flannigan et al. effectively impossible to implement. So, we should use pulse repetition rate, that is still compatible with normal cryo-EM operations.

15. It would be helpful for the authors to provide a hypothesis why the previously reported advantages of pulsed illumination are not observed in this case, to clarify how their results relate to prior studies.

We thank the reviewer for the valuable feedback. We have made changes in the discussion section of the revised manuscript in lines 288- 314 to address the reviewer comments.

16. Please include EMPIAR deposition of the raw data for validation purposes.

The EMBL-EBI server EMPIAR requires the existence of an EMDB 3D volume reconstruction. Since this is not existing for the BR 2D crystal images, we instead uploaded the images to the BioImage Archive, which is also hosted by the EMBL-EBI. The data are available at the BioImage Archive under accession number S-BIAD2466 (https://doi.org/10.6019/S-BIAD2466)

This is mentioned in line 417-418.

Reviewer #1 (Remarks on code availability):

The code is concise and clear and appears to provide a transparent, end-to-end analysis pipeline. However, not finding the raw images, I was unable to run the code to verify its functionality.

We thank the reveiwer for positive comments. Based on reviewer comment, we have uploaded the raw images to the EMBL-EBI BioImage Archive, as listed above.

Reviewer #2 (Remarks to the Author):

The authors have presented a very important study of radiation damage in cryoEM, comparing an RF pulsed electron source to a continuous source of the same flux for biological specimens at liquid nitrogen temperatures. The work is well done and convincing, and will be very important to the field. I have only a few minor comments.

We thank the reviewers for the encouraging words.

1. In figure 1, the scale bar indicates that the electron lissajous pattern covers an area of about 2.3 x 3 micrometers. In the text on page 5 it says that a 50 micrometer aperture was inserted downstream of the raster pattern to chop the beam. How is this possible? Is the patten magnified before the aperture? Please indicate on the pattern in the figure how large the aperture that cuts it actually is. Please also clarify how the lissajous pattern was measured.

We thank the reviewer for this comment. As reviewer asked, we have added a small discussion to clarify this in the revised manuscript, which can be found in lines 347-359. Yes, the illuminated area on the sample is smaller than the aperture diameter. This demagnification is implemented through the C2, C3 and Objective-minilenses in the column.

2. On page 5 it says that 75% of the bunches were empty. Is this controlled only by the size of the aperture or also by the RF modulation parameters? Please explain how this ratio is controlled in detail.

We thank the reviewer for this comment. Based on reviewer comment, we have added a small discussion to clarify this in the revised manuscript, which can be found in lines 158-162.

3. The performance of the TEM without the RF enabled is far below what would be expected for a normal Krios with a cFEG, as indicated by the tests with a cross grating in Supplementary figure 2; compare this to the performance seen in ref 25. Please explain why the performance is so much worse?

We thank the reviewer for this comment. Based on reviewer comment, we have taken high-resolution images of gold grating, as shown in Supplementary Figure 5. The image shows no considerable loss in performance while using pulsed mode for taking high-resolution images.

Minor:

Perhaps "limiting" instead of "hindering" in the first sentence of the abstract.

Working on reviewer comment, we have modified the terminology in the revised manuscript.

Reviewer #2 (Remarks on code availability):

-

Reviewer #3 (Remarks to the Author):

This paper presents a systematic study of radiation damage on paraffin crystals, purple membrane crystals, and TMV crystals using a radio-frequency (RF) driven 300 kV Titan Krios microscope equipped with a cold field emission gun (c-FEG) to generate highly regular pulsed electron beams. The results demonstrate that pulsed-electron illumination does not reduce beam damage in imaging biological macromolecules. Overall, the experiments are well-designed and rigorous, and the conclusions provide valuable insights into the ongoing debate regarding whether pulsed illumination can mitigate sample radiation damage. However, the following issues should be reasonably resolved or explained before publication.

1) In Figure 1 of the manuscript, the authors only provide a schematic diagram of the TEM setup with brief descriptions. Could they supplement some experimental images or data to better illustrate the differences between the pulsed and random illumination modes?

We provide in Figure 1 a scheme of the setup, which also indicates that in pulsed mode we have short time windows in which electrons reach the sample, while in non-pulsed mode, brightness

was reduced so that roughly the same number of electrons per time reach the sample. Resulting real-space images look "normal" in both modes, as shown in Supplementary Figure 3.

Additionally, we have added a schematic figure in Supplementary information (Supplementary Figure 1), to better visualize the working of RF cavity to produce pulsed electron beam.

2) On page 5 of the manuscript, the authors state: "It is important to note that under typical experimental conditions, approximately 75% of the electron bunches were empty, while only about 25% contained single-electron events, as detailed in Supplementary Table 1." However, I could not find Supplementary Table 1 in the Supplementary Information.

We thank the reviewer for directing our attention to this point. We have added the Supplementary Table 1 in the revised manuscript.

3) Also on page 5, the authors mention: "For comparison, the temporal profile of a conventional electron beam is also shown, which was obtained by switching off the RF cavity and adjusting the condenser lens settings so that the same beam brightness and diameter was achieved." It's not sure whether "the temporal profile of a conventional electron beam" shown here refers to the content in Figure 1c. The authors should clearly indicate this, and clarify whether the result is experimental or theoretical.

We thank the reviewer for this comment. Based on reviewer comment we have provided detailed desciption in the caption of Figure 1. Also, we have descibed the experimental values for electron per pulse in the Supplementary Table 1.

4) The authors should label the (1,1) and (2,1) planes in Figure 2a to facilitate reader interpretation. Similar labeling should be applied in Figures 3 and 4.

We thank the reviewer for this suggestion and we have changed the figures accordingly in the revised manuscript.

5) In Figures 2, 3, and 4, the authors present numerous curves with error bars. When stating the Ne values obtained from these curves (e.g., 11.81 e−/Å², 12.19 e−/Å², 38.15 e−/Å², 35.22 e−/Å²), the error range should be included. Furthermore, the authors mention "The radiation damage profiles, obtained from 10 independent measurements under both pulsed and random illumination conditions." I suggest that the statistical data for each independent measurement be tabulated in the Supplementary Information.

We thank the reviewer for valuable feedback. We have listed the values in detail in Supplemntary information and made corrosponding changes in the revised manuscript, which can be found in lines 106-108, 236-237, and 257-258.

6) For paraffin crystals, the method for quantifying diffraction intensity is clear. However, for the purple membrane and TMV polycrystals shown in Figures 3 and 4, it is unclear how the diffraction

intensities were extracted and calculated under segmented conditions. Could the authors provide more detailed explanations?

We thank the reviewer for the feedback. We want to point out that in addition to providing details in the methods section of the main text, we have supplementary figures for describing the processing of each dataset, i.e., Supplementary Figure 7 for Paraffin, Supplementary Figure 9 for purple membrane and Supplementary Figure 11 for TMV. We have added more detail for the processing of purple membrane and TMV datasets in the Supplementary Information.

7) The authors note: "In our study, the interval between consecutive pulses was 13.33 ns, substantially longer than the 160 ps used by Kisielowski et al.18 or the 192 ps used by Choe et al.19." However, Flannigan et al. reported that radiation damage mitigation was observed at intervals of 5–20 μs between consecutive pulses. Did the authors conduct comparative experiments using different pulse intervals? What is their perspective on the influence of pulse interval on sample radiation damage?

We thank the reviewer for pointing us in this direction. First, in the current implementation of our RF cavity, we cannot change the time between pulses. Both cavities are driven at a fixed frequency of 75 MHz, which locks the pulse interval at 13.33 ns for all of our experiments.

As discussed in a previous study by Flannigan et al., it is generally believed that increasing the time between electron pulses enhances damage mitigation by allowing sufficient time for energy dissipation. In our study, the interval between pulses is 13.33 ns. Kisielowski et al. used a much shorter interval of 160 ps between pulses. Despite recording slower, we still do not observe any reduction in damage. Flannigan et al. used much longer pulse intervals (5, 20, and 100 µs), but this results in extremely slow dose delivery. For example, using the dose rate reported by Flannigan et al., it would take approximately 2.78×10^5 s (more than three days) to acquire a single image with a dose of 50 $e^-/Å^2$, which is not practical.

Even with current advances in high-brightness CFEGs that allow operation at higher beam currents, increasing the time between incoming pulses would still lead to unacceptably long acquisition times for a single micrograph. In practical cryo-EM workflows, hundreds to thousands of micrographs must be collected, making the approach demonstrated by Flannigan et al. effectively impossible to implement. So, while using bigger time bwteen pulses may lead to more damage reduction, but we have to use illumination conditions comptible with normal cryo-EM conditions.

8) The authors need to revise Ref. 20 in the manuscript to cite its formal version (The Innovation Life 3:100145).

Based on reviewer comment, the reference has been updated in the revised manuscript.

9) Based on their experiments, the authors ultimately conclude that "Pulsed-electron illumination does not reduce beam damage for imaging biological macromolecules." Regarding this controversial issue, have the authors discussed the underlying mechanisms responsible for their

experimental results? I suggest the authors provide some reasonable explanations and theoretical analysis for these findings, rather than solely presenting experimental conclusions.

We thank the reviewer for the critical reading of the manuscript. Based on reviewer comment, we have added a new discussion to explain why we don't observe improvement in the discussion section of the revised manuscript, which can be found in lines 288-314.

REVIEWER COMMENTS

Reviewer #1 (Remarks to the Author):

The revision resolved earlier ambiguities and provided details explanations of how the RF cavity selects signal and provided informative summary of previous pulsed illumination work. It also provided important SPA metrics, including B-factors, and the discussion of the challenges in acquiring SPA data is particularly informative for guiding future work. Only minor comments for the authors to consider address:

Supplementary Figure 5 is to support that "the RF cavity has no significant negative impact on the microscope's performance" but the absolute value of the signals in the two radial profiles seem to be different enough that need an explanation, for example the signal at 0.487 Å-1 for conventional illumination >~0.08 but that of pulsed illumination is ~0.015.

We thank this reviewer for the encouraging comments. We added an explanation for this in the legend of Supplementary Figure 5: The data for the pulsed mode were taken at lower defocus, resulting in lower intensity values for pulsed illumination.

Reviewer #2 (Remarks to the Author):

The authors have made many improvements to the manuscript that are much appreciated. This is an important paper.

There is one serious error remaining though. The new supplementary figure 5 now presents an excellent comparison of the effect of the pulsed vs. conventional TEM. It is clear from the FFTs (loss of Thon rings at high resolution and removal of gold rings along one axis) that there is severe degradation in the spatial coherence caused by RF cavity. This is as expected (by me, anyhow) and exactly the opposite of the statement "demonstrating that the RF cavity has no significant negative impact on the microscope's performance." in the caption. Please correct this and include a description of the loss of spatial coherence and increased tilt in the text. I do not think this changes any of the conclusions regarding the radiation damage measurements in the rest of the paper but it is essential to include this as part of evaluating the effect of the RF cavity on the source and imaging performance.

We thank the reviewers for the encouraging words.

Based on reviewer comment, we have modified the caption of the Supplementary Figure 5. But as the reviewer mentioned, this doesn't influence the conclusion of the paper regarding radiation damage. The new legend now reads:

Supplementary Figure 5. High-resolution TEM images of gold grating with different electron illumination (left), and corresponding Fourier transforms of the images (middle), showing

information transfer in different directions up to the Nyquist limit (Magnification: 195kx, Pixel size: 0.403 Å). Radial profiles (right) show for both, pulsed and conventional TEM illumination, information up to very high frequencies. The Image acquired with the pulsed mode shows partial loss of information in higher order diffraction rings 0.687 Å-1 (1.45 Å), and 8.07 Å-1 (1.24 Å), resulting from combination of increased beam tilt and loss of spatial coherence. The data for the pulsed mode were taken at lower defocus, resulting in lower intensity values for pulsed illumination.

Reviewer #3 (Remarks to the Author):

The authors have responded to my comments. Changes have been made to manuscript and supporting information in light of my comments, which improve the quality of paper. As such, I recommend publication in Nature Communications with further editorial check.

By the way, I noticed a possible error in the newly revised manuscript. Please pay attention to correcting it by the authors: Regarding the data provided in Supplementary Table 2, I observed that the average critical dose of Paraffin in the Pulsed and Random modes should be 11.15 and 11.71 respectively, rather than the 11.81 and 12.25 mentioned in the current text. Please carefully check the data in the manuscript.

We thank the reviewer for encouraging comments.

Also, we thank the reviewer for pointing out the error in the values for paraffin damage. We have made relevant changes in the revised manuscript.

REVIEWER COMMENTS

Reviewer #2 (Remarks to the Author):

The authors have again improved the manuscript. The beam alignments in Fig S4 are still not optimal in either case, making comparison difficult, but the new figure S5 makes it clear how much is lost. It is now good enough for publication.

We thank the reviewer for the positive assessment and acknowledge the alignment limitation in Figure S4. We are glad that Figure S5 clearly demonstrates the performance of pulsed illumination.
